# Comparative Morphology of the Lungs and Skin of two *Anura*, *Pelophylax nigromaculatus* and *Bufo gargarizans*

**DOI:** 10.1038/s41598-020-65746-y

**Published:** 2020-07-10

**Authors:** Gan Guangming, Yang Zhe, Zhuang Mei, Zhang Chenchen, Ding Jiawei, Zhang Dongyu

**Affiliations:** School of Medicine, Southeast University, Nanjing, 210009 China

**Keywords:** Animal physiology, Herpetology, Zoology, Anatomy

## Abstract

The lungs and skin are important respiratory organs in *Anura*, but the pulmonary structure of amphibians remains unclear due to the lack of a suitable procedure. This study improved the procedure used for fixing lungs tissues and used light microscopy, transmission electron microscopy and scanning electron microscopy to reveal the differences in the lung and skin morphologies between *Pelophylax nigromaculatus* (*P. nigromaculatus*) and *Bufo gargarizans* (*B. gargarizans*). In *P. nigromaculatus* and *B. gargarizans*, the cystic lungs comprise a continuous outer pulmonary wall on which primary, secondary, and tertiary septa attach, and a number of regular lattices form from raised capillaries and the pulmonary epithelium on the surfaces of the pulmonary wall and septa. Each lattice in *P. nigromaculatus* consists of several elliptical sheets and flat bottom, and the septa are distributed with denser sheets and have a larger stretching range than the pulmonary wall. The lattice in *B. gargarizans* consists of thick folds and an uneven bottom with several thin folds, and the septa have more developed thick and thin folds than the pulmonary wall. However, the density of the pulmonary microvilli, the area of a single capillary, the thicknesses of the blood-air barrier, pulmonary wall and septum, and the lung/body weight percentage obtained for *B. gargarizans* were higher than those found for *P. nigromaculatus*. In *P. nigromaculatus*, the dorsal skin has dense capillaries and a ring surface structure with mucus layer on the stratum corneum, and the ventral skin is slightly keratinized. In *B. gargarizans*, the stratum corneum in both the dorsal and ventral skins is completely keratinized. A fine ultrastructure analysis of *P. nigromaculatus* and *B. gargarizans* revealed that the pulmonary septa are more developed than the pulmonary walls, which means that the septa have a stronger respiratory function. The more developed lungs are helpful for the adaptation of *B. gargarizans* to drought environments, whereas *P. nigromaculatus* has to rely on more vigorous skin respiration to adapt to a humid environment.

## Introduction

Pulmonary respiration plays an essential role in terrestrial animals^1,2^, but lower amphibians^3–5^, including *Anura*, *Urodela* and *Apoda*, adopt different strategies to inhabit aquatic, terrestrial or fossorial environments through gas exchange^6–8^. In most adult amphibians, the lungs, skin, and buccal cavities are involved in gas exchange^9^. However, in some fossorial environments, active respiration is found in the skin and buccal cavity, and the structure of the lung is as simple as a smooth capsule in some urodeles^10^. In several extreme examples, some amphibians, including *Anura*, have no lungs^11–13^. In drought and high-temperature environments, pulmonary respiration has an absolute advantage^9^, but skin respiration, even that in keratinized skin^14,15^, only works during hibernation^5^.

Therefore, amphibians have different compatible respiratory organs to adapt to diverse environments, and the structure of the respiratory organs is highly variable and particularly prominent in the structure of the lungs and skin.

The lungs in most *Anura* are faviform, and the primary, secondary and tertiary septa together constitute scaffolds of stretchable faveoli that are similar to those of amphibians, as demonstrated by scanning and transmission electron microscopy (SEM and TEM, respectively)^6,10,16–22^, and capillaries that are densely distributed on the septum. Morphological studies have shown that amphibian lungs mainly cover with a single type of pneumocytes, and they combines features of flat type I and cubic type II alveolar cells of the mammalian lung^16–19^. Another important respiratory organ in amphibians is the skin^23^. The skin plays an important role in removing carbon dioxide^5^, and gas exchange in the skin accounts for approximately two-thirds of the total CO_2_ emissions from amphibians. Some studies have even indicated that in amphibians, skin respiration is more important than lung respiration^10^. In recent years, studies on the skin of amphibians have mainly focused on the glands for medical purposes^24–26^ and the studies as the organ highly adapted to the different environmental condition^5,9,25^.

*B. gargarizans*^27^ belongs to the *Bufo* genus, *Bufonidae* family and *Anura* order. *B. gargarizans* adults have a lot of convex scrofula on their dorsal skin. *B. gargarizans* is widely distributed in mainland China and is a typical terrestrial amphibian in China. *P. nigromaculatus*^28^ belongs to the *Pelophylax* genus, *Ranidae* family and *Anura* order. Adults of this species tend to live near ponds and grasses, and it is also widely distributed in mainland China and is a typical amphibious amphibian in China. Because the skin characteristics and habitat environments show marked differences between these two representative species, we hypothesized that the respiratory systems of the two species, mainly the lungs and skin, have corresponding adaptive characteristics and differences.

However, observing the physiological structure of appropriately inflated lungs is difficult without a suitable procedure, and comprehensive morphological studies on the lungs of amphibians using the same experimental method have not yielded sufficient results. Here, we improved the procedure for the fixing of moderately inflated lungs and utilized light microscopy (LM), TEM and SEM to investigate the differences in the lung and skin morphologies between *P. nigromaculatus*^27^ and *B. gargarizans*^28^ adults to reveal why *P. nigromaculatus* and *B. gargarizans* live in humid and drought environments, respectively.

## Materials and Methods

### Experimental animals

**Figure 1:**
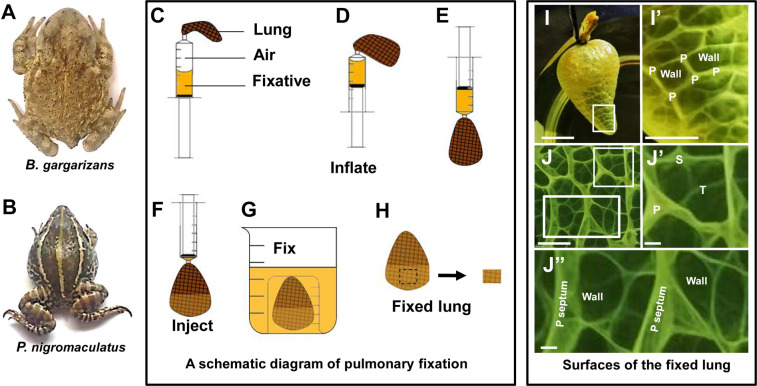
A schematic diagram of pulmonary fixation and the structure of inflated lung. *B. gargarizans* exhibits scrofulas on the dorsal skin (**A**), and *P. nigromaculatus* has smooth dorsal skin (**B**). Schematic diagram of pulmonary fixation (**C–H**). The lung sample is placed at the end of a syringe filled with air and fixative (**C**). Air is injected into the lung (**D**). The syringe is placed in a downward position (**E**). The fixative is injected into the lung (**F**). The lung is soaked in fixative using two beakers (**G**). The samples was then removed for experiments (**H**). Surfaces of the inflated lungs of *B. gargarizans* (**I-J**”) fixed with Bouin’s fixative. Outer surface of the fixed lung: the fixed lung contains the pulmonary wall and is divided into many grids by primary (P) septa (**I-I**’). Inner surface of the fixed lung: the lung has many honeycomb faveoli that consist of primary, secondary (**S**), and tertiary (T) septa and part of the pulmonary wall (**J-J**”). I’ shows enlarged images of the boxes in I, and J’ and J” show enlarged images of the white boxes in J. Scale bars, I: 1.5 cm; I’: 0.4 cm; J-J”: 1.5 mm.

Adult *B. gargarizans* (Fig. 1A) and *P. nigromaculata* (Fig. 1B) animals were collected in Nanjing, China, during the nonhibernating season and maintained in the Experimental Animal Center of Southeast University under 12-hour light/12-hour dark conditions at 25 °C^29^. All of the animals were bred in the animal facility at Southeast University, China, and all experimental protocols were approved by ethical committee/institutional review board of the Animal Care & Welfare Committee of Southeast University, China.

### “Inflate-inject” procedure for lung fixation

Amphibian lungs completely shrink after removal, which makes it difficult to observe the normal physiological structure of inflated lungs. We thus improved the procedure for fixing lung tissue (Fig. 1C–H).

First, a syringe from which the needle was removed was filled with 25 ml of fixative and 25 ml of air, and a lung was removed and attached to the end of the syringe with a cotton rope (Fig. 1C). The syringe was maintained with the tip pointing up and used to inject 25 ml of air into the lung to inflate it (Fig. 1D), and the fixative was then injected into the lung with the syringe while the tip was pointing down (Fig. 1E,F). The lung was then tied tightly with cotton rope, removed from the syringe, moved the position of the floating lung to effectively fix desired portion for observation, and placed in two beakers, and the inner and outer surfaces of most of the lung tissue were then soaked in fixative for an appropriate time (Fig. 1G). If the lung floated, the small beaker was held down with a heavy object. A small piece of lung tissue that was fixed on both sides was used for further experiments involving SEM, TEM and LM (Fig. 1H). For light and electron microscopy experiments, the lungs of a minimum of 3 animals were analyzed.

### Preparation of skin specimens for LM

*P. nigromaculatus* and *B. gargarizans* were maintained in an environment at 4 °C for several hours to sedate and anesthetize the skin, which would eliminate any possible interference from chemical anesthetics^29^. The experimental process was performed as previously described^29^. Briefly, *P. nigromaculata* and *B. gargarizans* were placed in a refrigerator at 4 °C for several hours. The dorsal and ventral skin was removed, flattened onto filter paper to prevent curl, fixed in Bouin’s fluid for 24 h and then dehydrated with a graded alcohol series. The specimens, including the skins and lungs, were then infiltrated and embedded in paraffin, cut into thin seriate slices of approximately 2–4 μm, stained with hematoxylin and eosin, and observed with an Olympus microscope (IX73).

### Preparation of specimens for TEM

The experimental process used to prepare the specimens for TEM was performed as previously described^29^. Briefly, *P. nigromaculata* and *B. gargarizans* were maintained at 4 °C for several hours. The dorsal and ventral skin was removed, flattened onto filter paper and fixed in solution consisting of 2% glutaraldehyde and 2% formaldehyde. Both the skin and lungs were rinsed in PBS solution, fixed with 1% osmium tetroxide, and stained with 2% aqueous uranyl acetate. The specimens were then dehydrated using an ethanol series, infiltrated in propylene oxide and embedded in Epon 812 (SPI Science), and ultrathin sections (90 nm thick) were cut with an ultramicrotome (Leica UC7). The grids were poststained with saturated uranyl acetate and lead citrate, examined with a Hitachi H-7650 electron microscopy and recorded with a Gatan 830 digital camera CCD.

### Preparation of specimens for SEM

Both the skin and lungs were fixed in a solution consisting of 2% glutaraldehyde and 2% formaldehyde for 24 hours, washed three times with PBS solution for 30 minutes, fixed with 1% osmium tetroxide for 2 hours, and rinsed twice in distilled water. The specimens were then dehydrated using an ethanol series (50%, 70%, 85%, 95%, 100%, and 100%; 20 minutes per wash), incubated twice in isoamyl acetate (15 minutes per incubation), dried in carbon dioxide to the critical point (EMITECH K850), and sprayed with gold (HITACHI-E1010). The skin and lungs were observed using a Hitachi S-3400 scanning electron microscopy.

### Lung/weight and heart/weight measurement procedures

*P. nigromaculatus* and *B. gargarizans* were maintained in an environment at 4 °C for several hours to sedate and anesthetize, and weigh the animal, then take out the lungs and heart and weigh them separately. At least 14 animals were analyzed in each data.

### Image acquisition and statistical analysis

The number, length, width, and area of the sheets as well as the area of the bottom of *P. nigromaculatus* were measured with ImageJ (NIH); the lengths of the thick and thin folds and the area of the bottom of *B. gargarizans* were also measured with ImageJ. Additionally, the density of the microvilli and capillaries, the thickness of the blood-air barriers, and the area of pulmonary capillaries in *P. nigromaculatus* and *B. gargarizans* were determined for comparative analysis. Finally, GraphPad Prism 5 was used for further analysis of the statistical data, and conclusions were then drawn.

## Results

### SEM analysis of the ultrastructural differences between the pulmonary wall and septum of P. nigromaculatus

*P. nigromaculatus* and *B. gargarizans* had one trachea and two bronchi but no bronchioles, and their lungs contained many honeycomb faveoli that were interconnected by primary, secondary, and tertiary septa and pulmonary walls^9^. Using the improved experimental procedure, we observed the structures of the inner and outer surfaces of the inflated lungs at specific physiological states. The analysis of the shape of the lung of *B. gargarizans* (Fig. 1I) revealed that the inflated pulmonary wall was divided into many quadrilateral spaces by the primary septa inside the lung (Fig. 1I-I’). The primary septa were the highest and formed main frames of honeycomb faveoli (Fig. 1J-J”). The tertiary septa were the lowest, and the secondary septa were the middle (Fig. 1J-J’). All the primary, secondary, and tertiary septa attached to the lung wall and were also connected to each other (Fig. 1J-J”). All the inner surfaces of the inflated lungs, including the septa and pulmonary walls, in *P. nigromaculatus* and *B. gargarizans* were easily observed by SEM utilizing our improved fixing method (Fig. 1I-J”).

**Figure 2:**
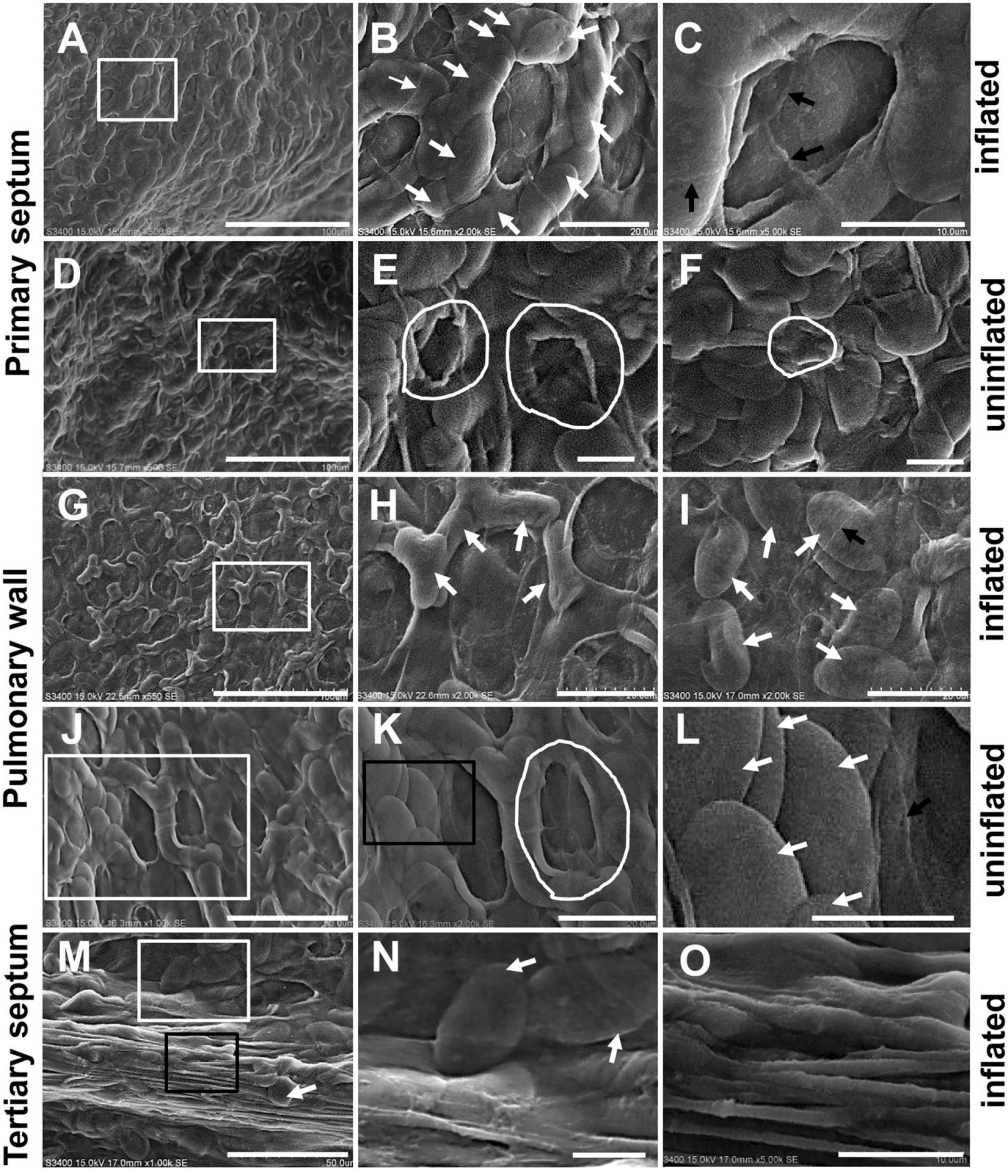
Ultrastructure of septum and pulmonary wall in *P.nigromaculatus* by SEM. SEM observation of the septa and pulmonary wall in *P. nigromaculatus*. In the primary septum surface of inflated lungs, the lattice includes sheets and a bottom (**A,B**), and the bottom is smooth (**C**). The uninflated lungs contain sheets and a bottom (**D,E**), and the overlapping sheets completely cover (**F**) or mostly (**E**) cover the bottom. Lattice, sparse sheets, and bottom in the pulmonary wall of inflated lungs (**G,H**). An inconspicuous lattice with a bottom and sheets (**I**) in the inflated lungs. The overlapping sheets (**J,L**) partially cover the bottom (**J,K**) in the pulmonary wall of uninflated lungs. The free end of the tertiary septa contains elongated filaments (**M,O**) and several sheets (**M,N**). B, E, H, K, and N show enlarged images of the white boxes in A, D, G, J, and M, respectively. L and O show enlarged images of the black boxes in K and M, respectively. The white thick arrow indicates a sheet, the black arrow shows the boundary of an epithelial cell, and the white curve shows the lattice in the uninflated lung. Scale bars, A, D, G: 100 μm; B, H-I, K: 20 μm; C, E-F, L, N-O: 10 μm; J, M: 50 μm.

In *P. nigromaculatus*, the primary septa and pulmonary walls were mainly observed due to the large surface area. The surfaces of capillaries formed regular annular morphology that we termed a fine lattice of septa and pulmonary walls in the inflated lungs, and the lattice contained several elliptical sheets and a flat bottom (Fig. 2A–L). In the primary septum, lattices were found around several partially overlapping elliptical sheets (Fig. 2A,B), and the bottom was located in the center of the lattice (Fig. 2B,C). The sheets were actually protruding capillaries that would be subsequently identified by TEM. In the septum of the uninflated lung, the lattices, which also included elliptical sheets and a bottom, were retracted, and the sheets were disordered and overlapped with each other (Fig. 2D–F); additionally, the bottom was significantly reduced (Fig. 2D,E) or even occluded (Fig. 2D,F) in the uninflated lattice, which resulted in lattices that could not be easily distinguished.

The pulmonary walls of inflated lungs exhibited similar elliptical sheets and bottom in the lattice (Fig. 2G–I). However, the sheets in the septa (Fig. 2A,B) were far more developed than those in the pulmonary walls (Fig. 2G,H). The sheets were reduced (Fig. 2G,H) and became discontinuous (Fig. 2G,H) in contrast to the lack of an obvious bottom with several sheets (Fig. 2I). Furthermore, the lattice showed an obvious difference in the pulmonary wall between uninflated (Fig. 2J–L) and inflated lungs (Fig. 2G,H). The bottom in the pulmonary walls of uninflated lungs remained open (Fig. 2J,K) and was larger than the septa with overlapping (Fig. 2L) sheets, which showed that the septa had strong scalability. Bare sheets (Fig. 2M,N) and elongated filaments (Fig. 2O) were observed on the free end of the tertiary septa, which showed that the septa had a strong potential to extend. Furthermore, obvious boundaries of epithelial cells were found in both the sheets (Fig. 2C,I) and bottom (Fig. 2C,L).

**Figure 3:**
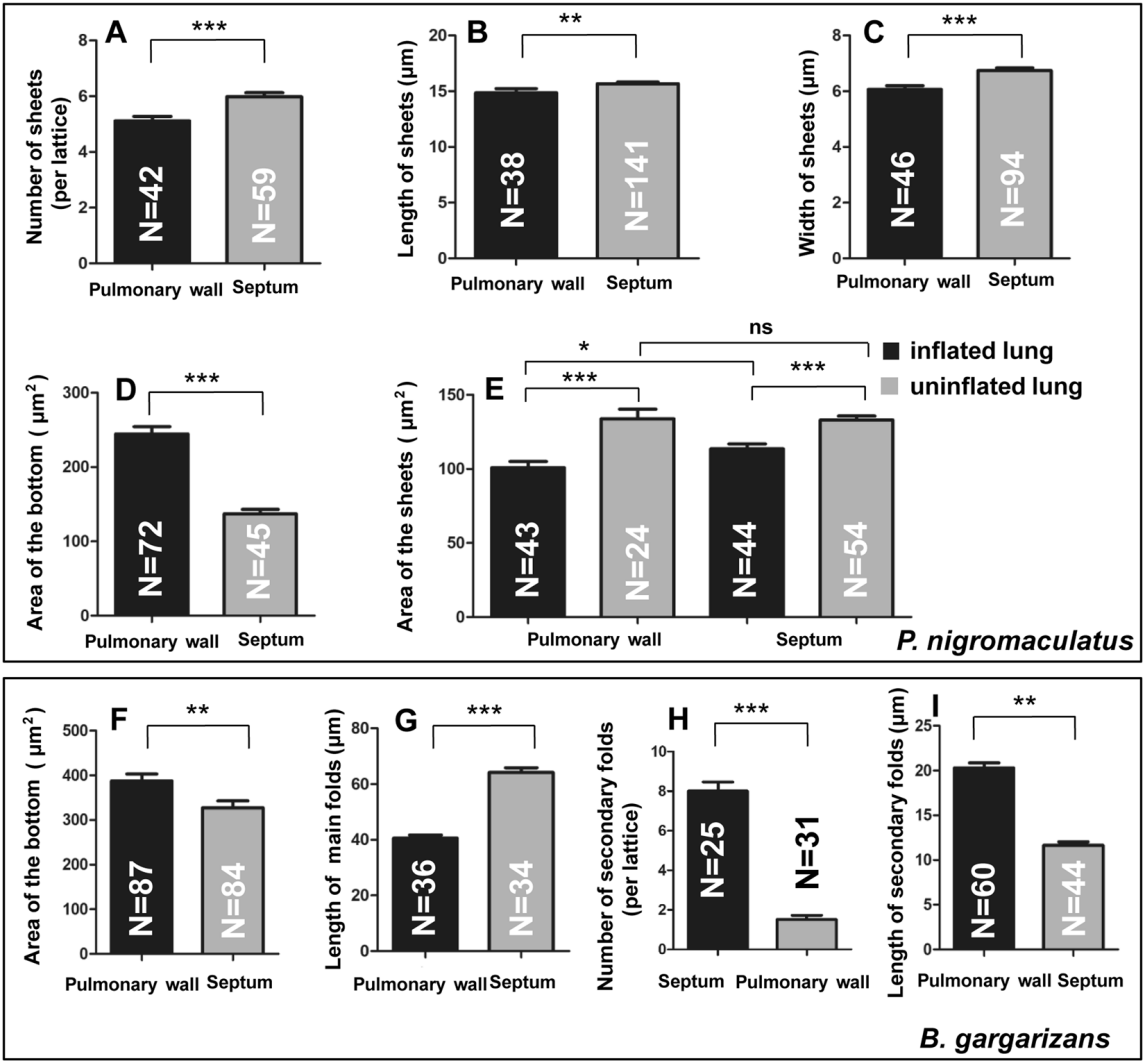
Comparative analysis of the pulmonary wall and septum in *P. nigromaculatus* and *B. gargarizans*. Number (**A**), length (**B**), and width (**C**) of the sheets in the pulmonary wall and septa in the inflated lung. Area of the lattice bottom in the pulmonary wall and septa in inflated lungs (**D**). Area of the sheets in the pulmonary wall and septa in both inflated and uninflated lungs (**E**). Area of the lattice bottom (**F**), length of the thick folds (**G**), and number (**H**) and length (**I**) of the thin folds in inflated lungs. The error bars indicate the SEMs, t test, two-tailed, *p < 0.05, **p < 0.01, ***p < 0.001.

Because the sheet was actually the surface of the protruding capillary that was subsequently verified by TEM and LM, we performed a quantitative comparison of the pulmonary walls and primary septa, which would reveal more differences between the pulmonary walls and septa in *P. nigromaculatus*. In inflated lungs, the sheets of the septa (5.983 ± 0.1399, N = 59) (per lattice) were more abundant than those of the pulmonary walls (5.119 ± 0.1606, N = 42) (Fig. 3A), and the length (15.68 μm ± 0.1467 μm, N = 141) and width of the sheets (6.745 μm ± 0.0892 μm, N = 94) in the septa were significantly increased compared with those in the pulmonary walls (length, 14.86 μm ± 0.3543 μm, N = 38; width, 6.063 μm ± 0.1327 μm, N = 46) (Fig. 3B,C). However, the area of the bottom in the pulmonary wall (244.5 μm^2^ ± 9.781 μm^2^, N = 72) was larger than that in the septa (137.4 μm^2^ ± 5.62 μm^2^, N = 45) (Fig. 3D) in inflated lungs, which showed that the lattice of the pulmonary wall had a stronger expansion ability than that of septa. A subsequent statistical analysis of the area of the sheets between uninflated and inflated lungs revealed that significant changes had occurred in both the pulmonary walls and septa (Fig. 2E). The areas of single sheets in both inflated pulmonary walls (100.8 μm^2^ ± 4.257, N = 43) and inflated septa (113.6 μm^2^ ± 3.384, N = 44) were smaller than those in uninflated pulmonary walls (133.9 μm^2^ ± 6.405, N = 24) and uninflated septa (132.9 μm^2^ ± 2.746, N = 54), which showed that the sheets and thus the surface areas of the capillaries were reduced during lung expansion. In inflated lungs, the area of a single sheet in septa was significantly larger (P = 0.024) than that in pulmonary walls (Fig. 2E), which showed that the surface areas of the capillaries was enlarged in the inflated state. However, no significant difference (P = 0.88) between septa and pulmonary walls was found in the contraction state (Fig. 2E), which showed that the capillaries of the pulmonary wall were expanded fully, regardless of the in inflated statues of the lung.

Therefore, based on the morphological structure of the lattice in *P. nigromaculatus* observed by SEM, the septa were more distributed and contained denser sheets (capillaries) than the pulmonary wall, and the sheets in the septa had a larger stretching range than the pulmonary wall. Since the area of the sheet actually represent the respiratory area that is covered by pneumocytes, so the respiratory function of pulmonary septa is more active than that of pulmonary wall in *P. nigromaculata*.

### SEM analysis of the ultrastructural differences between the pulmonary walls and septum of B. *gargarizans*

**Figure 4:**
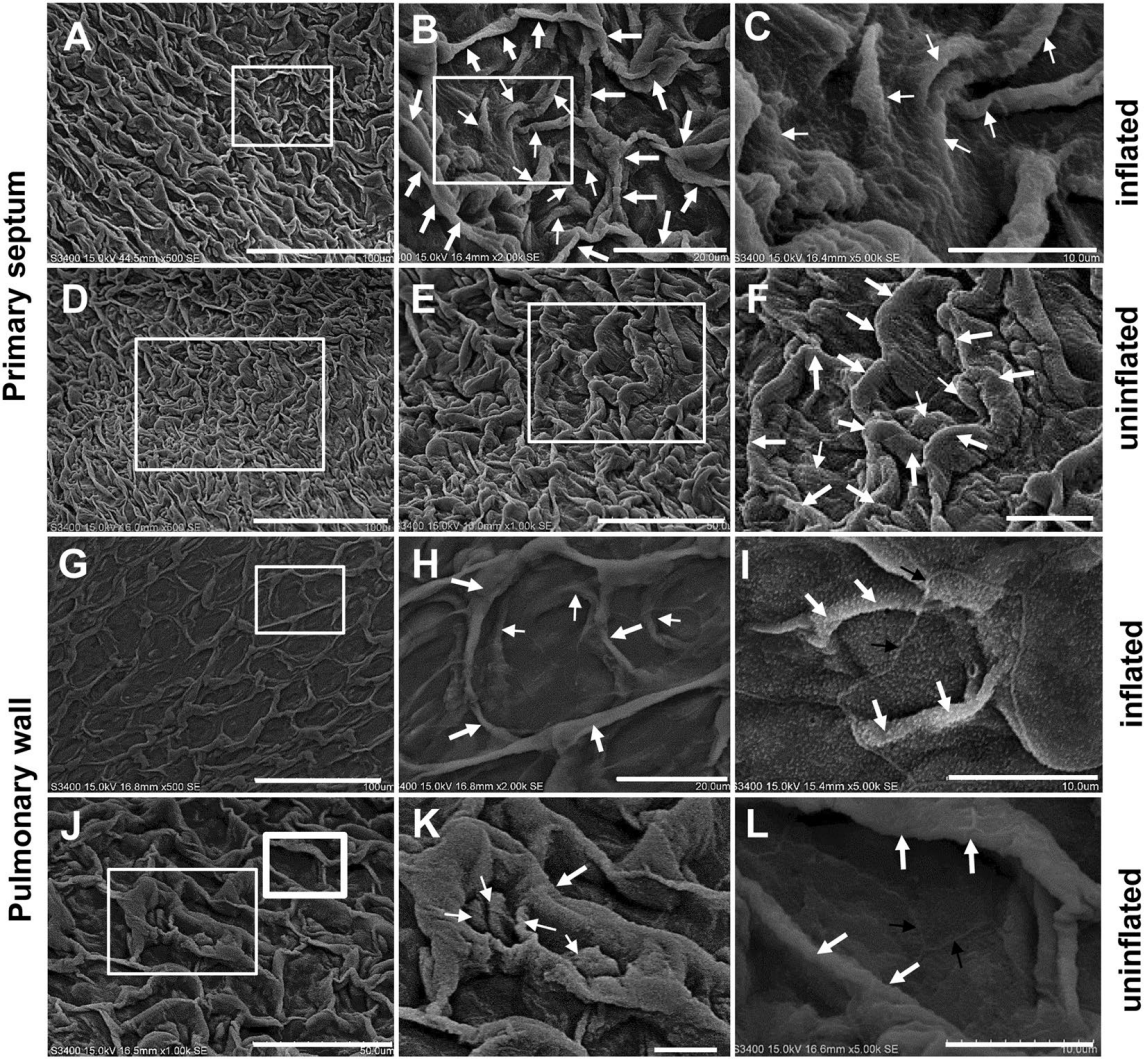
Ultrastructure of septum and pulmonary wall in *B. gargarizans* by SEM. SEM observation of the septa and pulmonary wall in *B. gargarizans*. The lattices include thick folds (**A,B**), thin folds (**B,C**) and the bottom (**B**) in the primary septa of inflated lungs, and the thin folds are located in the bottom (**B**). The uninflated lattices (**D,E**) contain thick folds (**F**) and thin folds (**F**) in the septa of uninflated lungs. Lattices (**G,I**), thick folds (**H**), spare thin folds (**H**) and absence of thin folds in the exposed bottom (**I**) in the pulmonary wall of inflated lungs. In the pulmonary wall of uninflated lungs, the lattices (**J,L**), thick folds (**K,L**) and sparse thin folds (**K**) shrink, becoming thick or spherical (**K**). B, C, E, F, H, and K show enlarged images of the boxes in A, B, D, E, G, and J, respectively. L shows an enlarged image of the thick boxes in J. The white thick arrow shows a thick fold, the white thin arrow indicates a thin fold, and the black arrow shows the boundary of an epithelial cell. Scale bar, A, D, G: 100 μm; B, H: 20 μm; C, F, I, K, L: 10 μm; E, J: 50 μm.

The honeycomb faveoli in *B. gargarizans* were similar to those in *P. nigromaculatus*, but the fine structure of the pulmonary walls and septa in *B. gargarizans* (Fig. 4) was different from that in *P. nigromaculatus*. The surface of the inflated lungs comprised polygonal lattices in both the septa (Fig. 4A–C) and pulmonary walls (Fig. 4G–I), and the lattice included the bottom and thick folds that were also subsequently confirmed to be capillaries. In the septa, the curved thick folds were distributed around the lattice, and the bottom was located in the center of the lattice (Fig. 4A,B). Notably, many curved thin folds were observed in the bottom (Fig. 4B,C). In the septa of uninflated lungs, the lattices were retracted (Fig. 4D,E), and the thick and thin folds and bottom maintained an uninflated state (Fig. 4E,F). The morphological structure of the pulmonary wall (Fig. 4G–L) was similar to that of the septa (Fig. 4A–F), but in inflated lungs, the lattices, including the thick folds and spare thin folds (Fig. 4G,H), were extremely stretched linearly, and the bottom was revealed (Fig. 4G,H) in the pulmonary walls. Moreover, the thin folds appeared to be sparse or absent (Fig. 4I). The lattices had a relatively regular shape (Fig. 4J), and the thick and thin folds in the pulmonary walls of uninflated lungs shrank, becoming thick (Fig. 4J,K) or spherical (Fig. 4J,L); in addition, the thin folds in the bottom were sparsely distributed (Fig. 4K) or absent (Fig. 4L), which also showed that the pulmonary wall had a stronger expansion ability than the septa. Similar to the results obtained for *P. nigromaculatus*, clear boundaries of epithelial cells were found in the thick (Fig. 4I) and thin folds (Fig. 4K) and bottom (Fig. 4K,L). In uninflated lungs, the thin folds contracted in the septa (Fig. 4F) and pulmonary wall (Fig. 4K) and were not easily distinguished from the thick folds based on the morphology.

The area of the bottom, the length of the thick folds, and the number and length of the thin folds were then determined as indicators of the respiratory function in the pulmonary walls and septa of inflated lungs. The areas of the bottoms in the pulmonary walls (391.2 μm^2^ ± 11.58 μm^2^, N = 87) were much larger (P = 0.0012) than those in the septa (337.2 μm^2^ ± 11.54 μm^2^, N = 84) (Fig. 3F). The length of the thick folds (capillaries) in the pulmonary walls (40.54 μm ± 1.176 μm, N = 36) was markedly shorter than the length of the thick folds in the lattice of the septa (64.21 μm ± 1.691 μm, N = 34) (Fig. 3G). In inflated lungs, the thin folds of the septa (8.00 ± 0.466, N = 25) (per lattice) were higher in number than those of the pulmonary walls (1.52 ± 0.217, N = 31) (Fig. 3H), which meant that the bottom of the lattice has a larger respiratory surface area. Although the thin folds were smaller, the length of the thin folds in the pulmonary walls (20.31 μm ± 0.558 μm, N = 60) was markedly longer than that in the septa (11.64 μm ± 0.397 μm, N = 34) (Fig. 3I).

Therefore, based on the morphological structure observed by SEM, the septa had longer thick folds in the lattice and thinner thin folds on the bottom of the lattice compared with the pulmonary wall in *B. gargarizans*, which indicated that a single lattice on the septa of the lungs had a larger respiratory surface area than that of pulmonary wall both in *P. nigromaculatus* and *B. gargarizans*.

### Differences in capillaries between P. *nigromaculatus* and B. *gargarizans*

**Figure 5:**
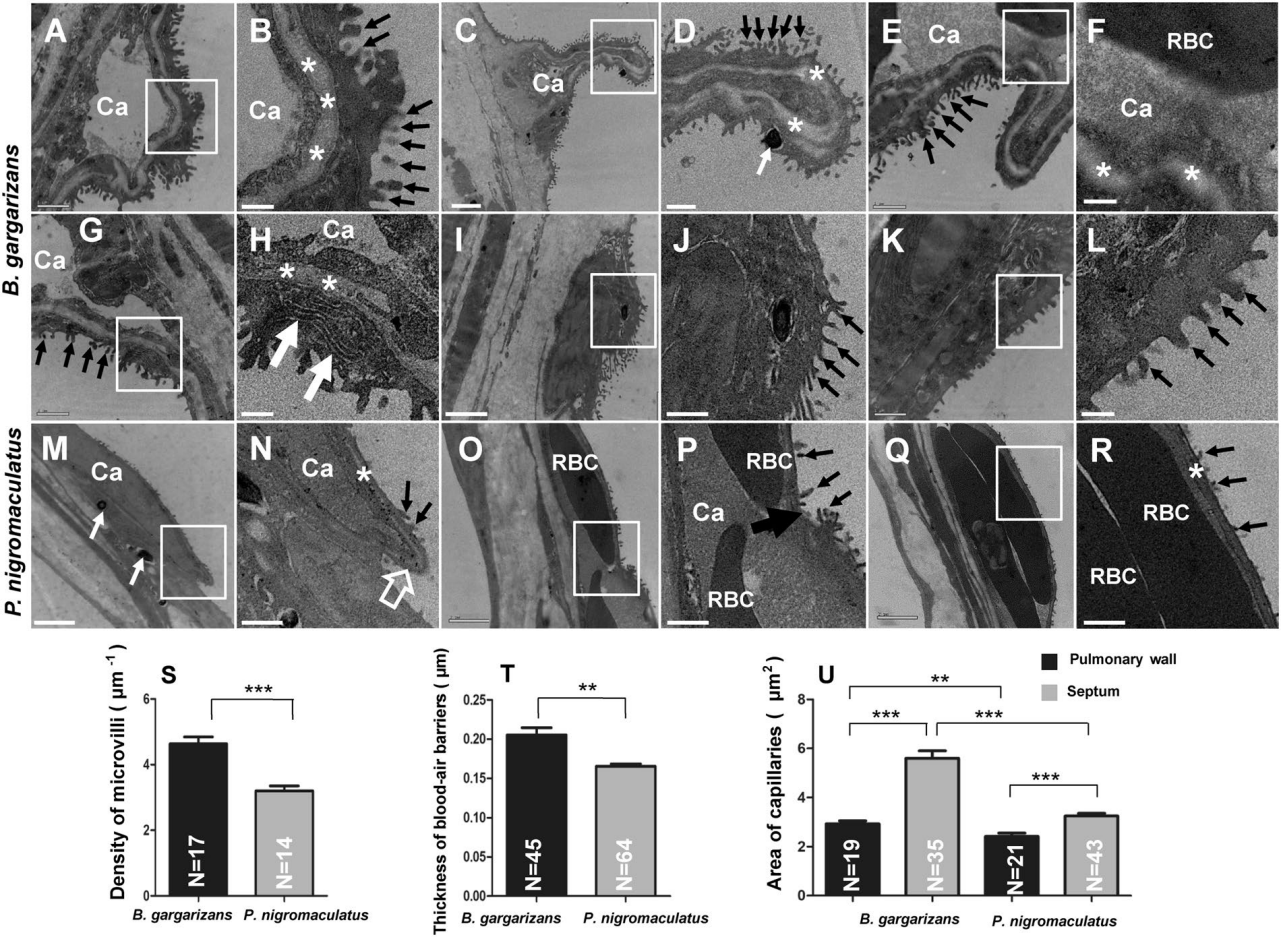
Ultrastructure of pulmonary capillaries in *P. nigromaculatus* and *B. gargarizans* by TEM. TEM observation of the pulmonary capillaries in *P. nigromaculatus* and *B. gargarizans*. TEM observations of the structures of the capillaries in *B. gargarizans* (**A–L**) and *P. nigromaculatus* (**M–R**). The capillary is located in a protruding thick fold (**A,B**) and under the basement membrane (*). The capillary (Ca) might swell (**A,B**) or shrink (**C,D**). The red blood cells (RBCs) (**E,F**) do not fill the capillaries. Endoplasmic reticulum and microvilli (**G,H**, white thick arrow). The thin fold does not contain capillaries (**I,J**). Dense microvilli in thin folds (**K,L**). The capillary is located under a sheet with sparse microvilli and a thin basement membrane (**M,N**). Sharp corner (white thick arrow) at the edge of the sheet (**N**). The two sheets interconnect and form a slight dent (**O,P**, black thick arrow). The capillaries contain one (**O,P**) or two layers (**Q,R**) of RBCs that fill the capillaries (**Q,R**). B, D, F, H, J, L, N, P, and R show enlarged images of the boxes in A, C, E, G, I, K, M, O, and Q, respectively. The white thin arrow shows melanosomes, and the black thin arrow indicates microvilli. Scale bar, A, E, G, K, M: 1 μm; C, I, O, Q: 2 μm; B, F, H, L, N: 0.35 μm; D, J, P, R: 0.7 μm. Density of microvilli (S), thickness of the blood-air barriers (T), and area of capillaries (**U**) in the inflated lungs of both *B. gargarizans* and *P. nigromaculatus*. The error bars indicate the SEMs, t test, two-tailed, *p < 0.05, **p < 0.01, ***p < 0.001.

According to the TEM analysis, the capillaries (Fig. 5A–E) of the lungs were located in the thick folds (Fig. 4) of the lattice in *B. gargarizans*. The thick folds prominently bulged into the lung cavity containing a dark cytoplasm of faveolar epithelium and dense microvilli^9^ as well as a thick basement membrane on the free surface (Fig. 5A,B); additionally, the capillary was located inside the basement membrane and formed the main body of the thick fold (Fig. 5A,B). The capillary might swell (Fig. 5A,B) or shrink (Fig. 5C,D), but the thick folds were protruding with dense microvilli, a thick basement membrane (Fig. 5A–D), and a pigment granule (Fig. 5D). Furthermore, the capillary contained red blood cells that did not fill the lumen of the capillary (Fig. 5E,F). Organelles, such as the endoplasmic reticulum, were detected between the basement membrane and the microvilli (Fig. 5G,H), which showed that the endothelium in the lung of *B. gargarizans* had a stronger ability to stretch. The thin fold was dark with dense microvilli, did not contain capillaries (Fig. 5I,J), and appeared as a cytoplasmic accumulation of faveolar epithelium with melanosomes (Fig. 5I,J). Dense microvilli were also present in relatively flat areas on the thin fold of the lattice (Fig. 5K,L).

According to the TEM observations, the capillaries of the lungs were located in the lattice sheets in *P. nigromaculatus*. The sheets contain melanosomes (Fig. 5M) that gently bulged into the lung cavity with sparse microvilli and a thin basement membrane (Fig. 5M,N) and formed a sharp corner at the edge of the free surface (Fig. 5N), which was also the edge of the sheets observed in the SEM images (Fig. 2A,B). The sheets interconnected, and a slight dent formed between two sheets (Fig. 5O,P). The capillaries contained one to two layers of red blood cells that filled the lumen of the capillaries (Fig. 5O–R) or did not contain red blood cells (Fig. 5M,N).

We compared the density of the microvilli, the thickness of the blood-air barriers and the area of the capillaries in the lungs between *B. gargarizans* and *P. nigromaculatus*. The density of the microvilli in the lungs of *B. gargarizans* (4.638 μm^−1^ ± 0.211 μm^−1^, N = 17) was significantly higher than that in the lungs of *P. nigromaculatus* (3.196 μm^−1^ ± 0.153 μm^−1^, N = 14) (Fig. 5S). The blood-air barrier in the lungs of *B. gargarizans* was thicker (0.205 μm ± 0.009 μm, N = 45) than that in the lungs of *P. nigromaculatus* (0.165 μm ± 0.003 μm, N = 64) (Fig. 5T), which showed that the efficiency of gas exchange in the lungs of *P. nigromaculatus* was higher than that in *B. gargarizans* but suggested that the capillaries in *B. gargarizans* had greater expansion potential than those in *P. nigromaculatus*. Furthermore, the area of single capillaries in the septum (*B. gargarizans*: 5.604 μm^2^ ± 0.301 μm^2^, N = 35; *P. nigromaculatus*: 2.937 μm^2^ ± 0.704 μm^2^, N = 19) was markedly larger than that in the pulmonary wall (*B. gargarizans*: 3.258 μm^2^ ± 0.109 μm^2^, N = 43; *P. nigromaculatus*: 2.425 μm^2^ ± 0.132 μm^2^, N = 21) (Fig. 5U). From another point of view, the areas of the capillaries in the pulmonary wall of *B. gargarizans* was larger (P = 0.07) than that in the pulmonary wall of *P. nigromaculatus*. Additionally, the area of the capillaries in the septum of *B. gargarizans* was notably larger than that in the septum of *P. nigromaculatus* (Fig. 5U). These data indicated that the areas of the capillaries in the septa was larger than that in the pulmonary wall in both *B. gargarizans* and *P. nigromaculatus*, but the areas of the capillaries and the microvilli density obtained for *B. gargarizans* were higher than those found for *P. nigromaculatus*.

**Figure 6:**
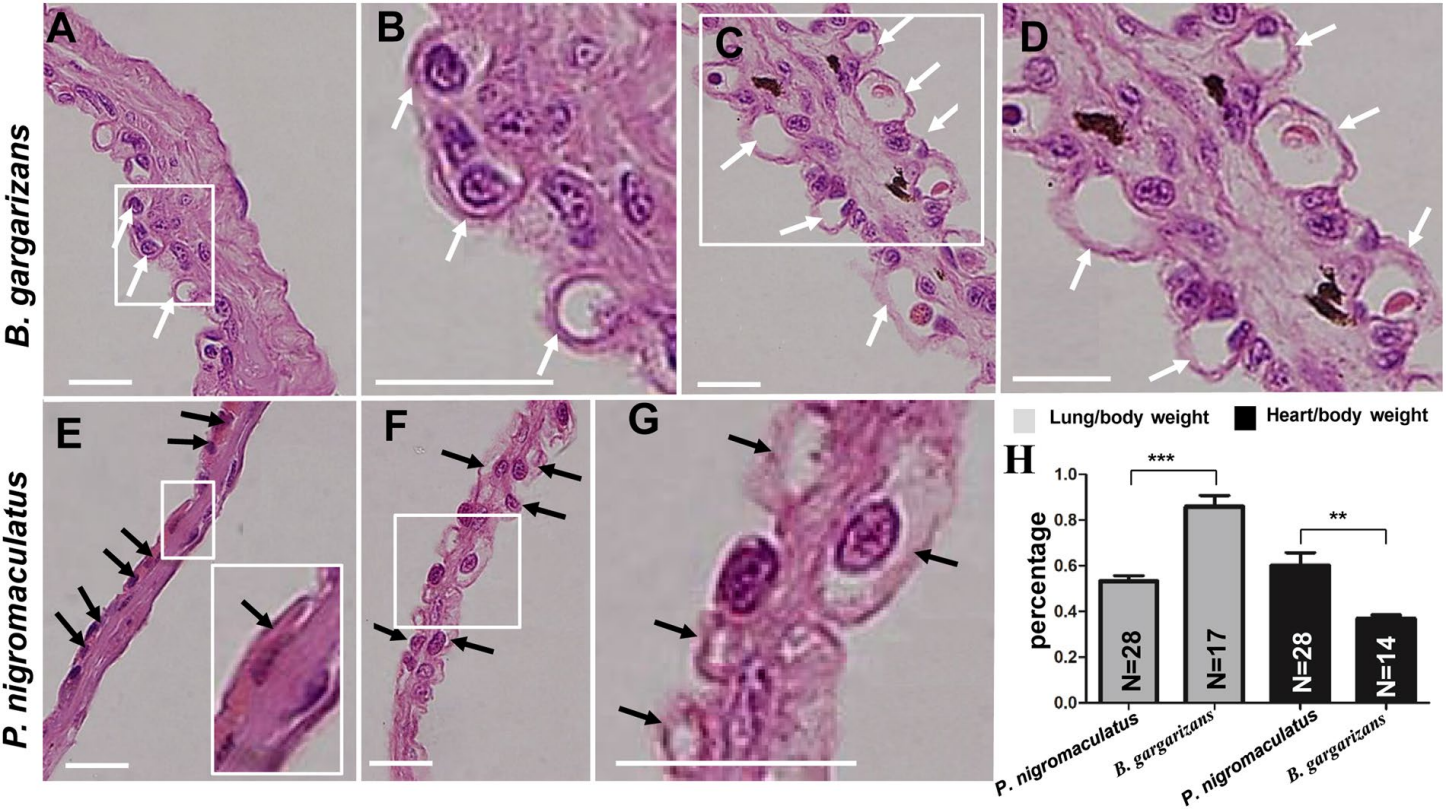
Structure of pulmonary capillary in *B. gargarizans* and *P. nigromaculatus* by LM. LM observation of the pulmonary capillaries in *B. gargarizans* and *P. nigromaculatus*. The pulmonary wall contains one layer of capillaries in *B. gargarizans* (**A,B**) and *P. nigromaculatus* (**E**) and is thicker in *B. gargarizans* (**A**) than in *P. nigromaculatus* (**E**). The septa contain two layers of capillaries in *B. gargarizans* (**C,D**) and *P. nigromaculatus* (**F,G**) and is thicker in *B. gargarizans* than in *P. nigromaculatus*. Lung/body weight percentage and heart/body weight percentages in *B. gargarizans* and *P. nigromaculatus* (**H**). The white arrow shows the capillary or thick fold of the lattice, and the black arrow shows the capillary or sheet of the lattice. B, D, and G show enlarged images of the boxes in A, C, and F, respectively. Scale bar: 30 μm.

The LM observations showed that the capillaries were distributed inside the pulmonary wall (Fig. 6A,B,E), but these structures existed on both sides of the septa in the inflated lungs of both *B. gargarizans* (Fig. 6C,D) and *P. nigromaculatus* (Fig. 6F–G). The thick folds of the lattice in *B. gargarizans* observed by TEM and LM were further confirmed as capillaries (Fig. 6A–D), and the lattice sheets in *P. nigromaculatus* detected by TEM and LM were also confirmed as capillaries (Fig. 6E–G). Moreover, the pulmonary wall (Fig. 6A,B) and septum (Fig. 6C,D) in *B. gargarizans* were markedly thicker than the pulmonary wall (Fig. 6E) and septum (Fig. 6F,G) in *P. nigromaculatus*, and the lung/body weight percentage obtained for *B. gargarizans* (0.86% ± 0.05) was notably higher than that in *P. nigromaculatus* (0.53% ± 0.03) (Fig. 6H). However, the heart/body weight percentage in *B. gargarizans* (0.37% ± 0.02) was substantially lower than that in *P. nigromaculatus* (0.60% ± 0.06) (Fig. 6H).

These data showed that the septa were the active area of lung respiration in *B. gargarizans* and *P. nigromaculatus* and further showed that the lung function in *B. gargarizans* was much stronger than that in *P. nigromaculatus*.

### SEM analysis of the different surfaces of the dorsal skin between *B. gargarizans* and *P. nigromaculatus*

**Figure 7:**
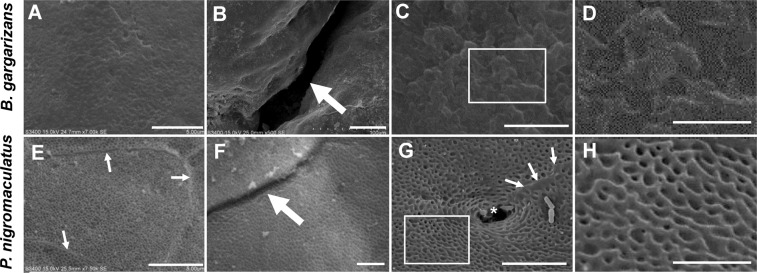
The surfaces of the dorsal skin in *B. gargarizans* and *P. nigromaculatus* by SEM. SEM observations of the dorsal skin in *B. gargarizans*, *P. nigromaculatus*. Horny surface (**A,C,D**) and shards (**D**) in the dorsal skin of *B. gargarizans*. (**B**) Deep groove of the dorsal skin, boundary among stratum corneum cells (**E,G**), shallow groove (**F**), and regular ring structure (**G,H**) in the dorsal skin of *P. nigromaculatus*. The white thick arrow shows the groove, and the white thin arrow shows the boundary among stratum corneum cells. D and H show enlarged images of the boxes in C and G, respectively. Scale bar, A, C, E, G: 5 μm; B, F: 50 μm; D, H: 2 μm.

*B. gargarizans* habituates environments with relative drought, whereas *P. nigromaculatus* has to live in humid environments. We predicted the existence of significant differences in the structure of the skin because the skin is another important respiratory organ in amphibians. We then analyzed the surface of the skin in *B. gargarizans* and *P. nigromaculatus* by SEM. The surface of the dorsal skin in *B. gargarizans* was covered by a scaly cuticle (Fig. 7A–D), and shards could be observed on the cuticle (Fig. 7C,D). We could not observe an obvious boundary among the stratum corneum cells, but a deep groove was detected in the skin (Fig. 7B).

However, in the dorsal skin of *P. nigromaculatus*, an obvious boundary was found among the stratum corneum cells (Fig. 7E,G), and the groove (Fig. 7F) between the stratum corneum cells was shallow. Moreover, the surface of the dorsal skin showed a regular ring structure (Fig. 7G,H) near an orifice of the gland, which would increase the contact area between the air and skin.

### SEM analysis of the different surfaces of the ventral skin between *B. gargarizans* and *P. nigromaculatus*

**Figure 8:**
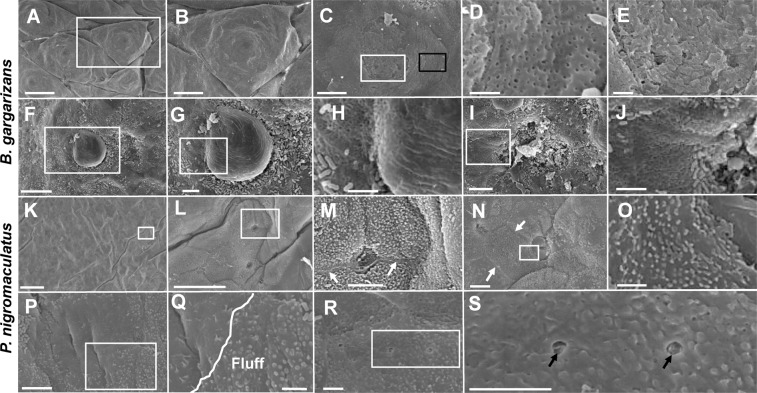
The surfaces of the ventral skin in *B. gargarizans* and *P. nigromaculatus* by SEM. SEM observations of the ventral skin in *B. gargarizans* and *P. nigromaculatus*. Surfaces of the ventral skin in *B. gargarizans* (**A–J**) and *P. nigromaculatus* (**K–S**). Plate in the ventral skins and granular gland (**A,B**). Ventral skin with superfine holes (**C,D**) and a scaly cuticle (**E**). Scale debris on skin with superfine holes (**F,H**). Superfine holes on the skin (**I,J**). Boundary among stratum corneum cells (**K–N**) with superfine fluff (**M–O**). Some fluff falls off and leaves a smooth surface (**P,Q**). The horny surface smashes, detaches and forms ultrafine pores (**R,S**). The white arrow shows the boundary among stratum corneum cells, and the black arrow shows the horny ultrafine pore. B, D, G, H, J, L, M, O, Q and S show enlarged images of the boxes in A, C, F, G, I, K, L, N, P and R, respectively. Scale bar, A: 500 μm; B: 250 μm; C, G, I, M, N, P, R: 5 μm; D-E, H, J, O, Q, S: 2 μm; F: 20 μm; K: 100 μm; L: 30 μm.

In *B. gargarizans*, the ventral skin consisted of many plates, and the granular gland was located in the center of the plate (Fig. 8A,B), as demonstrated by SEM. The skin with superfine holes (Fig. 8C,D) or that covered with scaly cuticle (Fig. 8C,E) would be exposed. The large pieces of scaly cuticle might shed along the circumference (Fig. 8F,G) of the skin with superfine holes (Fig. 8G,H). Once the scaly cuticle was completely detached, more superfine holes were exposed (Fig. 8I,J).

An obvious boundary was also found among the stratum corneum cells (Fig. 8K–N) in the ventral skin of *P. nigromaculatus*. No regular ring structure, such as that in dorsal skin (Fig. 7G,H), was observed, but a superfine adjunct that was considered to be keratinized fluff on the ventral skins was detected (Fig. 8L–O). The fluff gradually fell off and formed smooth skin (Fig. 8P,Q). The surface of stratum corneum cells would become smashed and would detach from the ventral skin, and form ultrafine pores (Fig. 8R,S).

### Different stratum cornea and subcutaneous capillaries in *P. nigromaculatus* and *B. gargarizans*

Because SEM only allows observations of the skin surface, we could not distinguish the skin characteristics between *B. gargarizans* and *P. nigromaculatus*. Therefore, further TEM and LM studies were performed to observe the skin structures of *B. gargarizans* and *P. nigromaculatus*.

**Figure 9:**
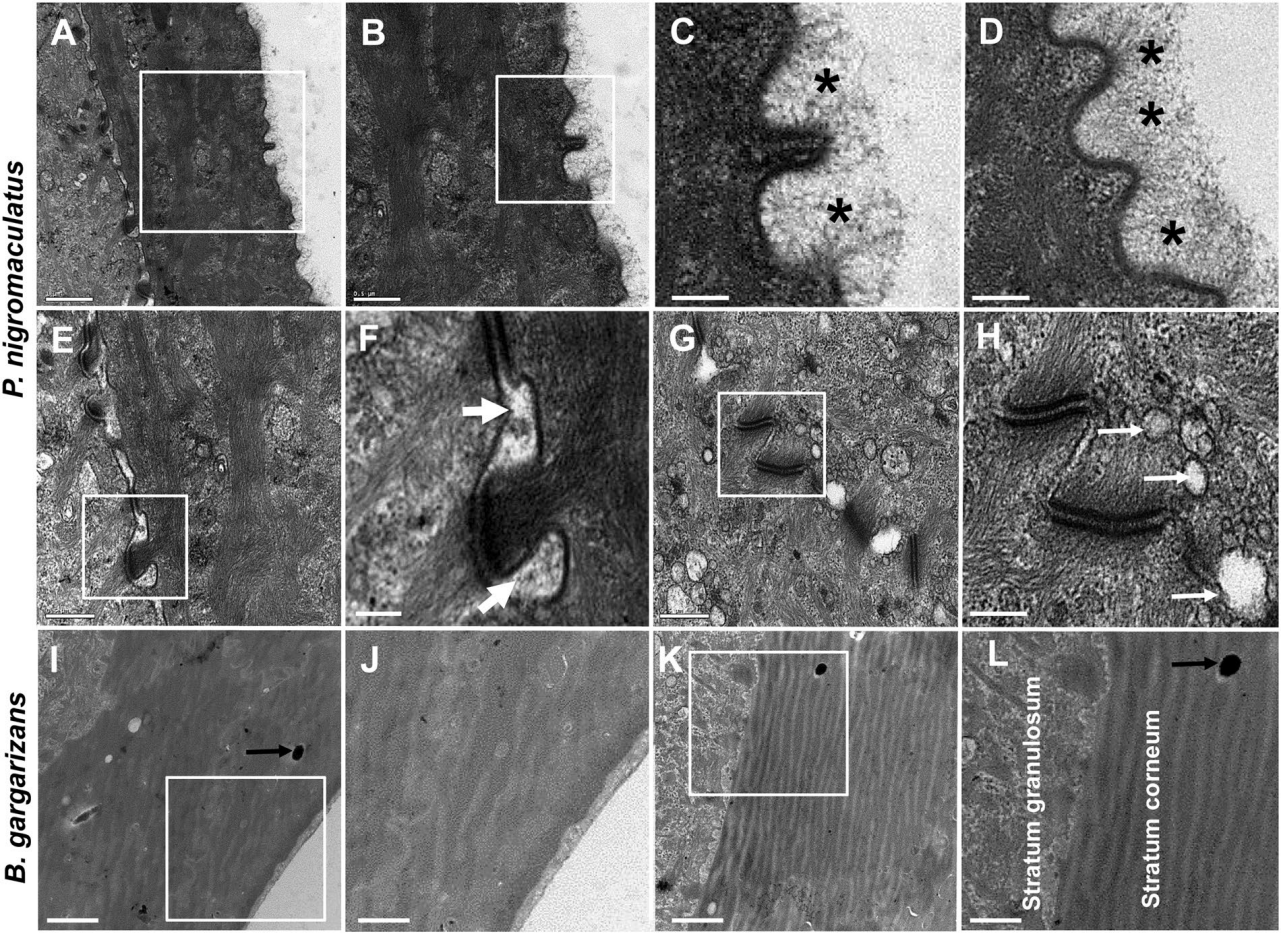
Ultrastructure of the dorsal stratum corneum in *P. nigromaculatus* and *B. gargarizans* by TEM. TEM observations of the dorsal stratum corneum in *P. nigromaculatus* and *B. gargarizans*. Nicks (**A–C**) and mucus layer (**B–D**, asterisk) in the surface of the dorsal skin in *P. nigromaculatus*. Degrading desmosome (**E,F**) with large separation bands and typical desmosomes (**G,H**) with bubbles (**H**). Smooth surface of thoroughly keratinized stratum corneum (**I,J**) and lack of cellular junctions (**K,L**) in the stratum corneum in *B. gargarizans*. The white thick arrow shows separation bands, and the white thin arrow shows a bubble. The small black arrow shows a pigment granule. B, C, F, H, J, and L show enlarged images of the boxes in A, B, E, G, I, and K, respectively. Scale bar, A, I, K: 1 μm; B, E, G,: 0.5 μm; C-D, F, H: 0.2 μm; J, L: 0.3 μm.

Many nicks (Fig. 9A–C) of different sizes were observed on the dorsal skin surface by TEM, and the nicks should be the superfine ring structure (Fig. 7G,H) that was observed by SEM. Moreover, in *P. nigromaculatus*, we observed a cottony mucus layer attached to the nicks by TEM in a high-power mode (Fig. 9A–D). The stratum corneum comprised several flattened keratinocytes, and desmosomes were distributed between two layers of keratinocytes (Fig. 9E–H). An obvious boundary line was detected between outer keratinocytes with degrading desmosomes (Fig. 9E,F), and the boundary line between inner keratinocytes with typical desmosomes and small bubble sizes was not obvious (Fig. 9G,H).

However, the stratum corneum was thoroughly keratinized and formed a multilayered fibrous structure (Fig. 9I–L) with melanosomes in *B. gargarizans*. The surface of the stratum corneum was smooth (Fig. 9I,J) and did not contain a mucus layer (Fig. 9I,J). The stratum corneum directly contacted the stratum granulosum without desmosomes (Fig. 9K,L). It appeared that the dorsal skin surface was active and suitable for gas exchange due to the presence of nicks and mucus layer detected by TEM and the superfine ring structure observed by SEM.

**Figure 10:**
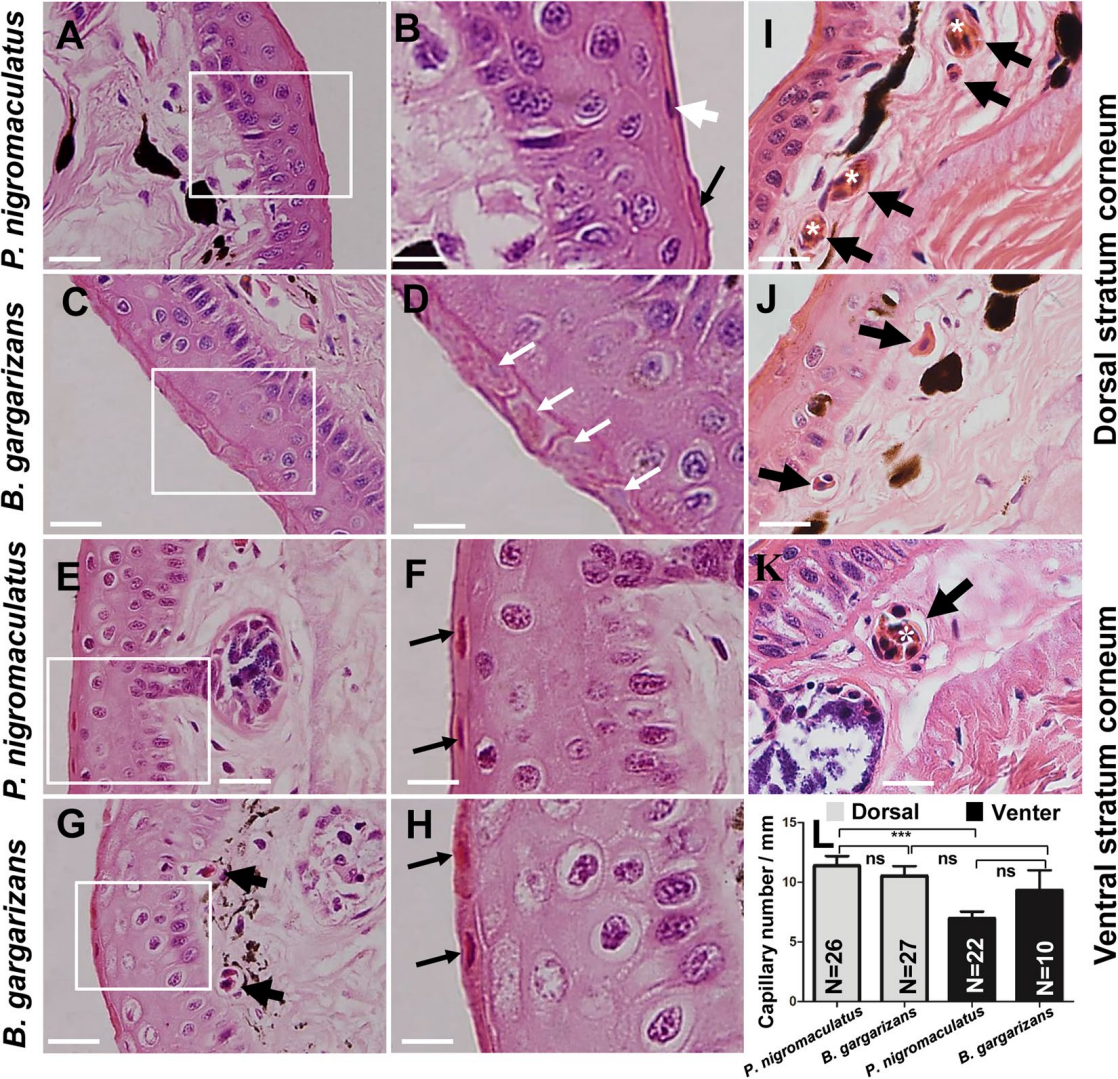
The stratum corneum and capillary in *P. nigromaculatus* and *B. gargarizans* by LM. LM observations of the stratum corneum and capillaries in *P. nigromaculatus* and *B. gargarizans*. The nucleus of the stratum corneum has a dark red or dark-purple-blue color (**A,B**) in the dorsal skin of *P. nigromaculatus* and almost completely disappears, leaving behind a light-blue residue (**C,D**), in *B. gargarizans*. The nucleus of the stratum corneum has a dark-red color in the ventral skin of *P. nigromaculatus* (**E,F**) and *B. gargarizans* (**G,H**). Capillaries in *P. nigromaculatus* (**I,J**) and *B. gargarizans* (**G,K**). Capillary density in *P. nigromaculatus* and *B. gargarizans* (**L**). B, D, F, and H show enlarged images of the boxes in A, C, E, and G, respectively. The white thick arrow shows a dark-purple nucleus, and the black thin arrow shows a dark-red nucleus. The white thin arrow shows the residue of the nucleus, and the black thick arrow shows a capillary. Asterisks show larger capillaries, which contain several red blood cells. Scale bar, A, C, E, G, I, J, K: 20 μm; B, D, F, H: 7 μm.

We observed the morphology of the nucleus by LM to further verify the physiological activity of different stratum cornea in *P. nigromaculatus* and *B. gargarizans*. The nuclei in the dorsal stratum corneum of *P. nigromaculatus* were fusiform and dyed either a dark-purple-blue or dark-red color (Fig. 10A,B) by hematoxylin. However, almost no nuclei were detected in the dorsal stratum corneum in *B. gargarizans*, which exhibited an obvious stratum corneum profile (Fig. 10C,D). In some stratum cornea, light-blue-colored traces of nuclei were observed, which indicated the nucleic acid that was released after nuclear lysis (Fig. 10C,D). The nuclei in the ventral stratum cornea of *P. nigromaculatus* and *B. gargarizans* were fusiform and dyed a dark-red color (Fig. 10E,F) (Fig. 10G,H), with hardly any dark-purple-blue color.

We subsequently assessed the density of the subcutaneous capillary network in *P. nigromaculatus* (Fig. 10I,J) and *B. gargarizans* (Fig. 10G,K). In *P. nigromaculatus*, the density of the dorsal capillary (11.38/mm ± 0.82, N = 26) was significantly higher than that in the ventral skin (6.97/mm ± 0.57, N = 22), but the density of the dorsal capillary (10.53/mm± 0.82, N = 27) was not significantly different from that of the ventral skin (9.32/mm ± 1.69, N = 10) of *B. gargarizans*. Although the density of the dorsal capillary and ventral capillary did not differ between *P. nigromaculatus* and *B. gargarizans* (Fig. 10L), *P. nigromaculatus* had larger capillaries, which could hold several red blood cells at the same time in dorsal skin (Fig. 10I) and ventral skin (Fig. 10K), while capillaries in dorsal skin (Fig. 10J) and ventral skin (Fig. 10G) of *B. gargarizans* only hold one red blood cell in most cases.

Thus, the dorsal stratum corneum had a main function in gas exchange in *P. nigromaculatus*, and the stratum corneum cells appeared to weakly support respiration in *B. gargarizans*.

## Discussion

### The “Inflate-Inject” procedure is helpful for fixation of the inflated lung of amphibians

Amphibians have cystic lungs with large internal spaces and very thin pulmonary walls. Once the cystic lungs of amphibians are removed from the body, they can easily undergo completely shrinkage or collapse unless filled with air before fixation, which is different from the findings obtained for the alveolar lungs of mammals, which contain many bronchioles and notably smaller internal spaces and are less likely to collapse.

The traditional method for lung fixation involves removal of the lungs, sampling a fragment and fixing the tissue sample in a fixative^30^ or only injection of a fixative into lungs, which are often uninflated^9^. In many cases, due to the presence of the bronchioles, the structure of the mammal lungs does not shrink substantially. However, because amphibians do not have bronchioles, these lung samples do not undergo physiological expansion. With our “Inflate - Inject” procedure, which involves “inflation” of the lung with air and the “injection” of fixative into the lung, we could observe the lungs under physiological expansion by injecting air and then fixing the tissue.

This method had several advantages. First, it was possible to obtain the fine ultrastructure of the amphibian lungs under moderately inflated conditions, which revealed the physiological pulmonary structure. The capillaries on the surface of the inflated lungs of *P. nigromaculatus* and *B. gargarizans* were arranged in a specific lattice. However, in uninflated lungs of *P. nigromaculatus*, the capillaries overlapped and showed a chaotic arrangement, and it was not easy to observe the shape and regularity of the capillaries, that were observed as sheets by SEM. Furthermore, the thin folds shrank into ellipsoid shapes in uninflated *B. gargarizans* lungs, as observed by SEM, and the shrunken thin folds could easily be mistaken for capillaries, but only the thick folds were capillaries. Therefore, the sheets in *P. nigromaculatus* and the thin folds in *B. gargarizans* were the protrusions of capillaries, which suggested that SEM, which has a wider field of view than TEM and paraffin sections, could be efficiently used to observe the capillaries in inflated lungs of amphibians, including *P. nigromaculatus* and *B. gargarizans*. Second, we found significant differences in the pulmonary ultrastructure of *P. nigromaculatus* and *B. gargarizans* using this method, and this method can also be used to treat reptilian lungs that do not contain bronchioles.

### The differences in fine pulmonary structure determine the corresponding function of pulmonary respiration in *P. nigromaculatus* and *B. gargarizans*

With the exception of some specialized tetrapods in which respiration is performed mainly via the skin^6–8,13^, most amphibians have elaborate lungs^31^ that are separated into many faveoli with dense capillaries. The large inner surface of the lung increases the gas exchange, and almost all amphibians contain microvilli^16,17,20^ that effectively increase the breathing area in the lungs, even in the inferior lungs of newt, which have a smooth sac and no septa^10^.

TEM, SEM and LM observations of the lung samples revealed that the sheets in *P. nigromaculatus* and folds in *B. gargarizans* were favorable for air exchange. However, the respiratory capacity of the septa was stronger than that of the pulmonary wall, and the exchange of air depended on the thick folds in *B. gargarizans* and sheets in *P. nigromaculatus* because these structures were actually the surface of the capillaries. The septa had a large surface area for the attachment of many capillaries and dense microvilli on both sides and were highly expandable for the attachment of capillaries^16,17,20^. Moreover, the capillaries in the septa were densely distributed compared with those in the pulmonary wall, and this finding was obtained for both *P. nigromaculatus* and *B. gargarizans*. Therefore, according to a statistical analysis, septa had a stronger respiratory function than the pulmonary wall in *P. nigromaculatus* and *B. gargarizans*.

The air-blood barrier serves as a criterion for assessing the respiratory capacity of amphibians, reptiles and mammals, and mammalian lungs have a stronger respiratory capacity^6^. In addition, during the evolutionary history of these species, the lungs are crucial to oxygen acquisition in organisms that moved from the ocean to land^32^. The integrity of the blood-air barrier is essential for pulmonary gas exchange by preventing the return of blood to the interstitial and alveolar cavities^33^. The thickness of the blood-air barriers of *B. gargarizans* was larger than that of *P. nigromaculatus*, which indicated that *P. nigromaculatus* had a higher capacity for gas exchange.

However, this study provided additional powerful evidence of the respiratory function in *B. gargarizans*. The lung/body weight percentage was much higher, the septa and pulmonary wall were thicker, the pulmonary microvilli were denser in *B. gargarizans* compared with *P. nigromaculatus*, and thin folds were found in the bottom of the lattice in *B. gargarizans*, which showed that the lungs of *B. gargarizans* had a more powerful respiratory function. Moreover, in *P. nigromaculatus*, the capillaries resembled sheets in the SEM images, and a slight dent between two sheets was found by TEM; in contrast, the SEM analysis of *B. gargarizans* showed that the capillaries resembled thick folds that help speed up blood flow. The TEM analysis of *B. gargarizans* revealed that the area of the capillaries was larger and that the red blood cells did not fill the capillary lumen, which was favors the movement of red blood cells, but the TEM analysis of *P. nigromaculatus* revealed that the red blood cells filled the capillary lumen. Therefore, a structural analysis of *P. nigromaculatus* demonstrated that the capillaries of the lungs were not conducive to blood flow. To ensure gas exchange in the lungs, a higher heart/body weight percentage should enhance blood flow, and a thinner gas-blood barrier should increase the gas exchange efficiency in *P. nigromaculatus*.

### The structure of the differentiated skin determines the corresponding function of the skin in *P. nigromaculatus* and *B. gargarizans*

Skin respiration is one of the important strategies used by amphibians to maintain their oxygen pressure and hydrogen ion balance^34^. Amphibians, which have two modes of breathing, freely adjust the proportion of lung and skin breathing according to the external environment^35,36^. As one of the important respiratory organs of amphibians, the respiratory ability of skin has mainly been evaluated by studying the blood flow in the skin^37^. Amphibians mainly breathe through the skin and gills before metamorphosis^5,38^. Additional studies have found that *Xenopus laevis* tadpoles develop much slower in water with a normal oxygen content than they do in air^39^. The amount of oxygen involved in skin breathing equals approximately 10% of the total oxygen demand^40^.

Observations of the dorsal skin revealed that the dorsal skin was covered with smooth horny pores in the stratum corneum of *B. gargarizans*, whereas *P. nigromaculatus* had many small regular ring structures and a mucus layer in the dorsal skin. These results indicated that the dorsal skin of *B. gargarizans* tended to protect, whereas that of *P. nigromaculatus* tended to breathe. Moreover, the SEM and TEM analyses of *P. nigromaculatus* revealed that the dorsal skin contained regular ring structures and was covered with mucus layer, respectively, and fusiform nuclei and desmosome connections were observed in the stratum corneum. In contrast, the skin of *B. gargarizans* was smooth and completely covered with keratinized stratum corneum without desmosome connections and few deformed nuclei. Furthermore, the capillary density of the dorsal skin was significantly larger than that of the ventral skin in *P. nigromaculatus*, which showed that the dorsal skin was more breathable than the ventral skin. These results indicated that the skin of *P. nigromaculatus* had physiological activity for breathing and that the skin of *B. gargarizans* was “dead” and had little function for breathing. This finding was similar to the phenomenon in which *P. nigromaculatus* reduces the ventilation of the lungs by floating on the water surface, thereby increasing the gas exchange rate in the skin and preventing hypoxia^36^.

However, we observed that the ventral skin was slightly keratinized and contained superfine fluff in some stratum cornea in *P. nigromaculatus*. Therefore, the ventral skin in *P. nigromaculatus* tended to protect in addition to its respiratory function due to the presence of hole-like structures^41^.

### Ethics approval and consent to participate

All of the animals were bred in the animal facility at Southeast University, China. All of the animals were bred in the animal facility at Southeast University, China. All of the experiments were performed according to the guidelines approved by Southeast University. All experimental protocols were approved by ethical committee/institutional review board of the Animal Care & Welfare Committee of Southeast University, China.

## Supplementary information

Supplementary Information.
