# Supplementary material for: Comparative Morphology of the Lungs and Skin of two Anura, Pelophylax nigromaculatus and Bufo gargarizans
Source: Sci Rep. 2020 Jul 10;10:11420. doi: 10.1038/s41598-020-65746-y (PMC7351734; doi:10.1038/s41598-020-65746-y)
Supplement: Supplementary file 1 — Supplementary Information. [file 41598_2020_65746_MOESM1_ESM.docx]

**Supplementary data**

The improved “Inflate-Inject” procedure is helpful for observing the morphological structure of the lung in amphibians. The capillaries are arranged in a number of regular lattices on the surfaces of the pulmonary wall and septa in *P. nigromaculatus* and *B. gargarizans*, as demonstrated by SEM. Each lattice in *P. nigromaculatus* consists of several elliptical sheets and flat bottoms (Fig. S1A-B), and each lattice in *B. gargarizans* contains thick folds and uneven bottoms with several thin folds (Fig. S1A-B). The septa in *P. nigromaculatus* and *B. gargarizans* have a stronger respiratory function with strong scalability and a large surface lattice that attaches to two layers of capillaries.

We proposed a pattern for the comparison of pulmonary lattice extension between *P. nigromaculatus* and *B. gargarizans*. *P. nigromaculatus* increased the respiratory area through the sheets and bottom (Fig. S1A-B), but the sheets could not expand fully and would overlap in inflated lungs (Fig. S1B). However, *B. gargarizans* increased the area through thin folds and thick folds (Fig. S1C-D), which could expand to a large extent and did not overlap even in uninflated lungs (Fig. S1C). Moreover, the thick folds were conducive to blood flow. In *B. gargarizans*, the capillaries can be more outstretched, and the respiratory epithelium on the surface of the lattice bottom with more thin folds might be more scalable. Therefore, pulmonary respiration was dominant in *B. gargarizans*, but *P. nigromaculatus* has to rely on respiratory dorsal skin that attaches dense capillaries, an extracellular matrix, and an uneven stratum corneum surface.


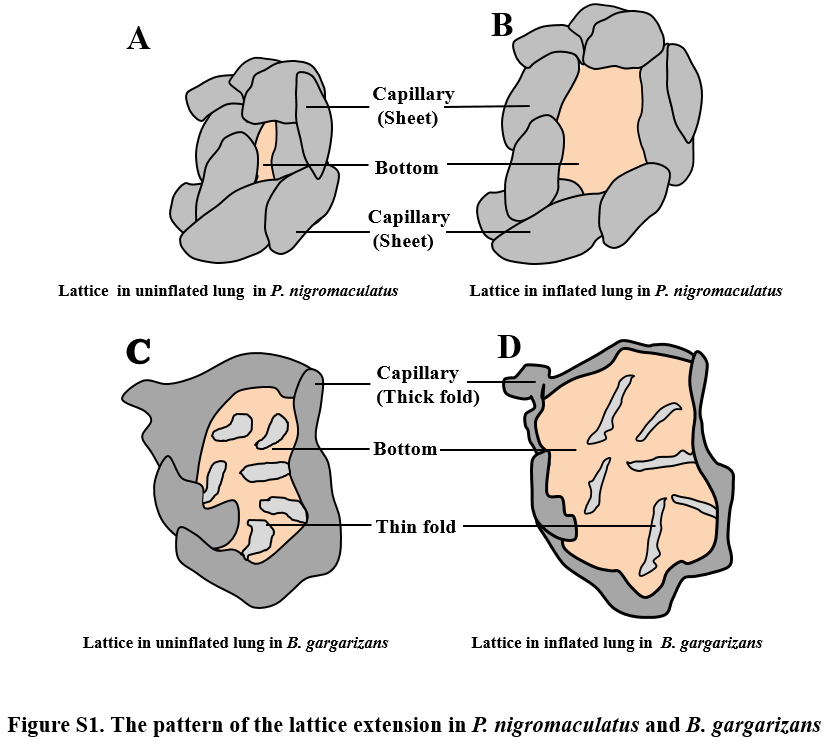


Capillaries arrange into lattices with sheets and bottom. (A) Lattice in uninflated lung in *P. nigromaculatus*, the sheets (capillaries) most overlap each other and almost obstruct the bottom; (B) Outstretched lattice in inflated lung in *P. nigromaculatus*, the sheets less overlap, and the bottom is fully extended; Capillaries arrange into lattices with thick folds and bottom on which there are several thin folds in *B. gargarizans*. (C) Lattice, include thick folds, bottom and thin folds, withdraw in uninflated lung. (D) Thick folds, bottom and thin folds expands in lattice in inflated lung.
